# The Anti-Microbial Peptide (Lin-SB056-1)_2_-K Reduces Pro-Inflammatory Cytokine Release through Interaction with *Pseudomonas aeruginosa* Lipopolysaccharide

**DOI:** 10.3390/antibiotics9090585

**Published:** 2020-09-08

**Authors:** Lucia Grassi, Arianna Pompilio, Esingül Kaya, Andrea C. Rinaldi, Enrico Sanjust, Giuseppantonio Maisetta, Aurélie Crabbé, Giovanni Di Bonaventura, Giovanna Batoni, Semih Esin

**Affiliations:** 1 Department of Translational Research and New Technologies in Medicine and Surgery, University of Pisa, 56123 Pisa PI, Italy; lucia.grassi@ugent.be (L.G.); e.kaya@studenti.unipi.it (E.K.); giuseppantonio.maisetta@dps.unipi.it (G.M.); 2 Department of Medical, Oral and Biotechnological Sciences, and Center for Advanced Studies and Technology (CAST), “G. d’Annunzio” University of Chieti-Pescara, 66100 Chieti CH, Italy; arianna.pompilio@unich.it (A.P.); giovanni.dibonaventura@unich.it (G.D.B.); 3 Department of Biomedical Sciences, University of Cagliari, 09142 Monserrato CA, Italy; rinaldi@unica.it (A.C.R.); sanjust@unica.it (E.S.); 4 Laboratory of Pharmaceutical Microbiology, Ghent University, 9000 Gent, Belgium; aurelie.crabbe@ugent.be

**Keywords:** anti-microbial peptide, dendrimeric peptide, *Pseudomonas aeruginosa*, LPS, anti-inflammatory activity

## Abstract

The ability of many anti-microbial peptides (AMPs) to modulate the host immune response has highlighted their possible therapeutic use to reduce uncontrolled inflammation during chronic infections. In the present study, we examined the anti-inflammatory potential of the semi-synthetic peptide lin-SB056-1 and its dendrimeric derivative (lin-SB056-1)_2_-K, which were previously found to have anti-microbial activity against *Pseudomonas aeruginosa* in in vivo-like models mimicking the challenging environment of chronically infected lungs (i.e., artificial sputum medium and 3-D lung mucosa model). The dendrimeric derivative exerted a stronger anti-inflammatory activity than its monomeric counterpart towards lung epithelial- and macrophage-cell lines stimulated with *P. aeruginosa* lipopolysaccharide (LPS), based on a marked decrease (up to 80%) in the LPS-induced production of different pro-inflammatory cytokines (i.e., IL-1β, IL-6 and IL-8). Accordingly, (lin-SB056-1)_2_-K exhibited a stronger LPS-binding affinity than its monomeric counterpart, thereby suggesting a role of peptide/LPS neutralizing interactions in the observed anti-inflammatory effect. Along with the anti-bacterial and anti-biofilm properties, the anti-inflammatory activity of (lin-SB056-1)_2_-K broadens its therapeutic potential in the context of chronic (biofilm-associated) infections.

## 1. Introduction

Anti-microbial peptides (AMPs) represent a key component of the innate immune system of many multicellular organisms, playing a major role in the first line of defense against a broad range of pathogens [1,2]. The anti-microbial properties of such peptides have been extensively studied over the last decade, and considerable effort has been made to develop them as novel therapeutic agents for the treatment of infections caused by antibiotic-resistant bacteria [3]. Nonetheless, in addition to the direct anti-microbial activity, many peptides have been found to exhibit multifaceted immunomodulatory properties at sub-inhibitory concentrations, including activation of controlled inflammatory responses, regulation of chemotaxis, modulation of cell differentiation, stimulation of angiogenesis and enhancement of wound healing [4,5]. All these activities contribute to the ability of AMPs to promote pathogen clearance and resolution of the infection, while preventing excessive and potentially harmful pro-inflammatory responses. For instance, the outcome of chronic infections (often biofilm-associated) is typically exacerbated by the intense host pro-inflammatory response to the persistent bacterial stimulus, which can lead to progressive tissue damage and organ dysfunction. Chronic infection and inflammation are, for example, observed in *Pseudomonas aeruginosa* lung infections in cystic fibrosis (CF) patients [6]. Hence, due to their dual (anti-bacterial and immunomodulatory) mechanism of action, AMPs are gaining increasing interest for the management of chronic infections [7,8,9,10,11].

In our previous work, we explored different strategies to enhance the anti-bacterial and anti-biofilm activity of the semi-synthetic peptide lin-SB056-1 [12] in physiologically relevant, host-mimicking conditions that are known to interfere with the stability and efficacy of AMPs [13,14]. These include the presence of salts at physiological levels, proteases, macromolecules of the body fluids as well as host cells, which significantly affect the ability of AMPs to interact with bacterial membranes [15,16]. Notably, combination treatments and peptide dendrimerization were found to significantly improve the effectiveness of lin-SB056-1 in in vivo-like models representative of *P. aeruginosa* chronic lung infection [17,18]. In particular, the peptide used in combination with the chelating agent ethylenediaminetetraacetic acid (EDTA) demonstrated the ability to inhibit *P. aeruginosa* biofilm formation in an artificial sputum medium mimicking the lung environment of CF patients [17], and exerted a marked bactericidal activity against endogenous *P. aeruginosa* in the sputum of primary ciliary dyskinesia (PCD) patients [19]. The anti-microbial and anti-biofilm activity of the peptide was further enhanced by modifying its structural properties, i.e., design of the dendrimeric derivative (lin-SB056-1)_2_-K. In particular, the dendrimeric peptide was found to exhibit 4 to 16-fold lower MIC (Minimal Inhibitory Concentration) values in cell culture medium (i.e., 2.4 to 9.6 µM) compared to lin-SB056-1 against both a reference strain and CF lung isolates of *P. aeruginosa* [18]. When tested in an in vivo-like 3-D lung epithelial cell model [13], (lin-SB056-1)_2_-K displayed a stronger anti-biofilm activity compared to both lin-SB056-1 and the lin-SB056-1/EDTA combination, causing a reduction of as much as 99.9% in the number of biofilm-forming bacteria at the concentration of 19.25 µM [18]. Based on these promising results, in the present study, lin-SB056-1 and its dendrimeric derivative were evaluated in terms of anti-inflammatory properties. Due to the crucial role of the lipopolysaccharide (LPS) in the induction of inflammation by Gram-negative bacteria [20], we investigated the ability of both peptides to inhibit LPS-elicited production of pro-inflammatory cytokines along with the mechanisms underlying their effect. The obtained results revealed the superiority of the dendrimeric peptide (lin-SB056-1)_2_-K over its monomeric counterpart in reducing cytokine production by lung epithelial- and macrophage-cell lines stimulated with *P. aeruginosa* LPS. In addition to the remarkable anti-microbial properties, the enhanced anti-inflammatory effect of the dendrimeric derivative underlined its broader therapeutic potential in the context of *P. aeruginosa* chronic lung infections.

## 2. Results

### 2.1. The Dendrimeric Peptide (Lin-SB056-1)_2_-K Significantly Reduces Cytokine Production by Lung Epithelial Cells and Macrophages Stimulated with LPS and/or P. aeruginosa

In order to determine the anti-inflammatory potential of lin-SB056-1 and its dendrimeric derivative (lin-SB056-1)_2_-K, we evaluated the effect of both peptides on the production of pro-inflammatory cytokines by lung epithelial cells (A549 cell line) and macrophage-like cells (i.e., THP-1 cells differentiated with 12-*O*-tetradecanoylphorbol-13-acetate, TPA) in response to *P. aeruginosa* LPS. In the case of A549 cells, we also exploited whole *P. aeruginosa* (PAO1 strain) as pro-inflammatory stimulus with the aim of investigating the ability of (lin-SB056-1)_2_-K to neutralize the effect of bacterial membrane-associated LPS molecules in addition to that of purified (soluble) LPS. Importantly, the bacterial inoculum used for the experiments contained a comparable amount of LPS (approximately 100 ng for 5 × 10^6^ CFU of *P. aeruginosa* PAO1) to that of soluble LPS (i.e., 160 ng). In order to avoid cell cytotoxicity induced by live bacteria, *P. aeruginosa* was fixed with paraformaldehyde as this treatment has been previously reported to preserve bacterial morphology and surface ultrastructure [21].

**Figure 1:**
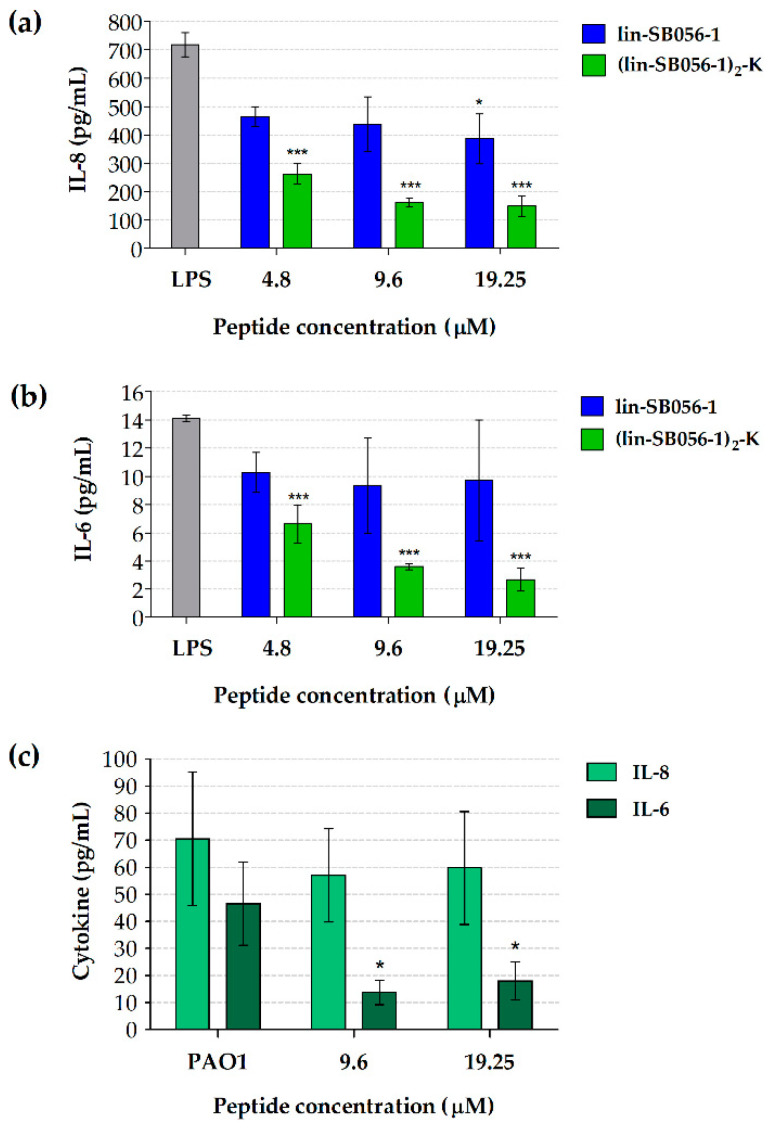
Cytokine production by A549 cells stimulated with purified *P. aeruginosa* LPS (**a**,**b**) or whole *P. aeruginosa* (**c**) in the presence of lin-SB056-1 and/or its dendrimeric derivative for 4 h (37 °C, 5% CO_2_). Production of IL-8 (**a**) and IL-6 (**b**) by A549 cells stimulated with LPS (800 ng/mL) in the presence of lin-SB056-1 and (lin-SB056-1)_2_-K. (**c**) production of IL-6 and IL-8 by A549 cells stimulated with *P. aeruginosa* PAO1 (fixed with 4% paraformaldehyde; 5 × 10^6^ CFU/well) in the presence of (lin-SB056-1)_2_-K. LPS: LPS-stimulated A549 cells without peptide treatment. PAO1: *P. aeruginosa*-stimulated A549 cells without peptide treatment. Data are reported as mean ± standard error of the mean (SEM) of at least three independent experiments. * *p* < 0.05, *** *p* < 0.001 compared to the peptide-untreated control (one-way ANOVA for paired samples followed by Tukey–Kramer post-hoc test).

In LPS-stimulated A549 cells, a significant reduction in the release of both IL-8 and IL-6 was observed in the presence of (lin-SB056-1)_2_-K (Figure 1a,b), while only a decrease in IL-8 levels was recorded in the presence of lin-SB056-1 at the highest concentration tested (19.25 μM; Figure 1a). In particular, the dendrimeric peptide was able to reduce IL-8 and IL-6 levels as much as 80% already at the concentration of 9.6 μM (Figure 1a,b). Interestingly, the same concentration of (lin-SB056-1)_2_-K determined a statistically significant reduction in IL-6 levels (up to 70%) in culture supernatants of A549 cells even when whole *P. aeruginosa* was employed as pro-inflammatory stimulus (Figure 1c). Conversely, no evident effect of (lin-SB056-1)_2_-K on IL-8 release by A549 cells was recorded (Figure 1c).

**Figure 2:**
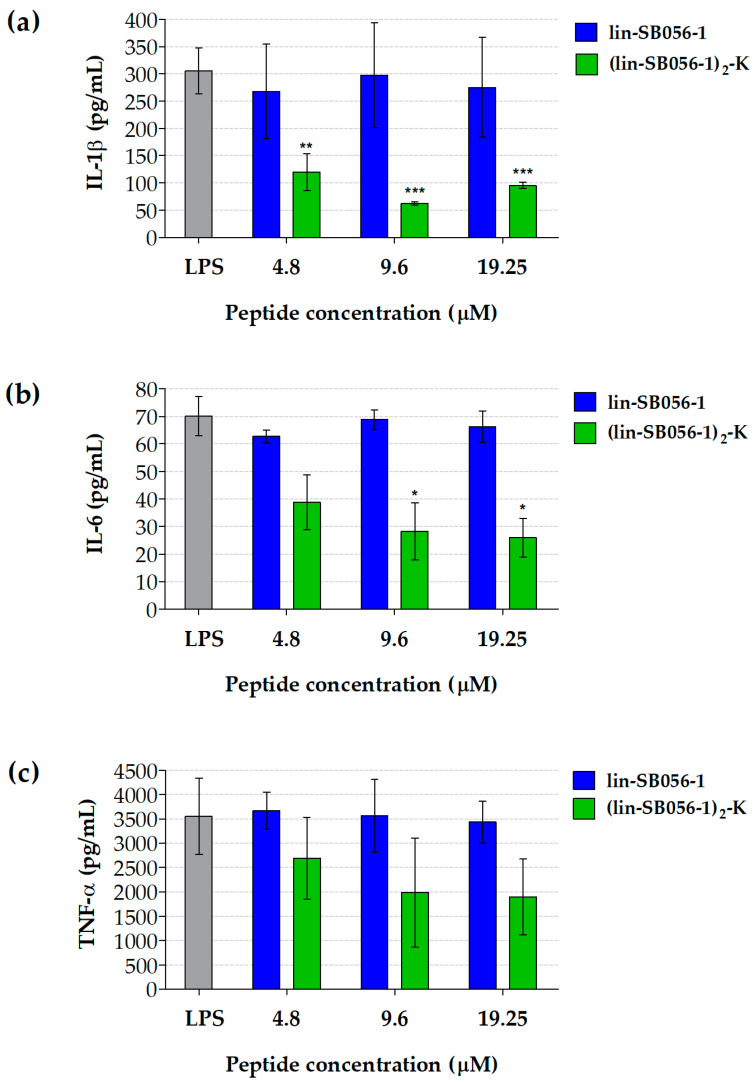
Production of IL-1β (**a**), IL-6 (**b**) and TNF-α (**c**) by macrophage-like cells (THP-1 macrophages) stimulated with purified *P. aeruginosa* LPS (10 ng/mL) in the presence of lin-SB056-1 and (lin-SB056-1)_2_-K for 4 h (37 °C, 5% CO_2_). LPS: LPS-stimulated THP-1 macrophages without peptide treatment. Data are reported as mean ± SEM of three independent experiments. * *p* < 0.05, ** *p* < 0.01, *** *p* < 0.001 compared to the LPS-stimulated (peptide-untreated) control (one-way ANOVA for paired samples followed by Tukey–Kramer post-hoc test).

In the case of LPS-stimulated THP-1 macrophages, exposure to (lin-SB056-1)_2_-K at 9.6 μM resulted in a statistically significant reduction in the secretion of both IL-1β (80%) and IL-6 (60%) compared to the LPS-stimulated (peptide-untreated) control (Figure 2a,b). Although (lin-SB056-1)_2_-K showed a trend in decreasing TNF-α levels, no statistical significance was reached for this cytokine (Figure 2c).

### 2.2. Lin-SB056-1 and Its Dendrimeric Derivative Do Not Affect Cell Viability

**Figure 3:**
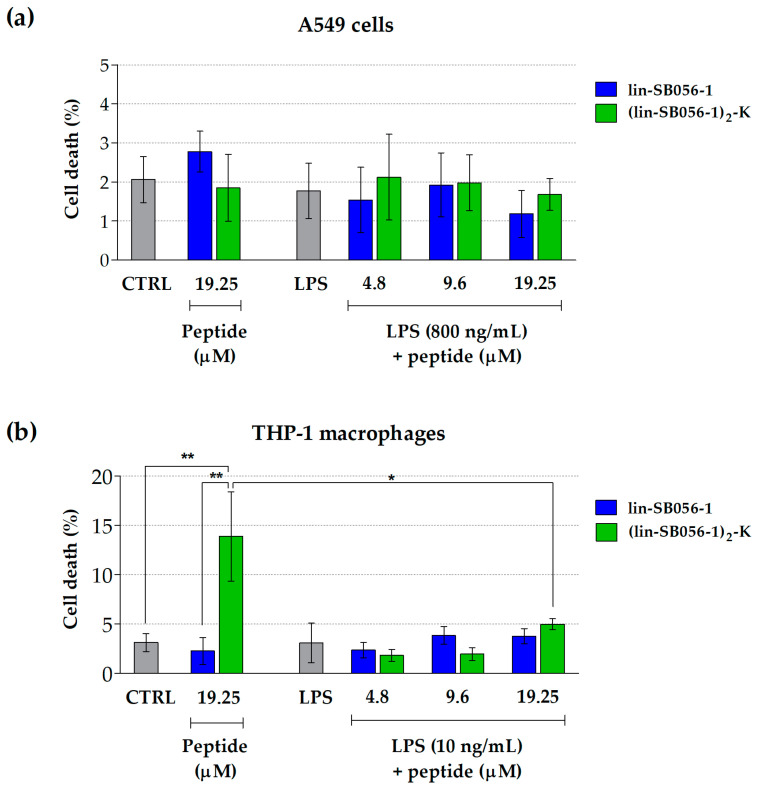
Cytotoxicity of lin-SB056-1 and (lin-SB056-1)_2_-K towards A549 cells (**a**) and THP-1 macrophages (**b**) after 4 h of incubation (37 °C, 5% CO_2_) in the presence or absence of *P. aeruginosa* LPS. CTRL: untreated cells. LPS: LPS-stimulated cells without peptide treatment. Data are reported as mean ± SEM of at least three independent experiments. * *p* < 0.05, ** *p* < 0.01 (Student’s *t*-test).

In order to exclude that the observed anti-inflammatory activity was due to a cytotoxic effect of lin-SB056-1 and (lin-SB056-1)_2_-K on the tested cell lines, we assessed the viability of A549 cells and THP-1 macrophages following LPS stimulation and peptide treatment. The cytotoxicity of both peptides at the maximum concentration used in the study (i.e., 19.25 μM) was also determined in the absence of LPS. Both peptides displayed no detectable toxicity (less than 5% cell death) towards LPS-stimulated A549 cells (Figure 3a) and THP-1 macrophages (Figure 3b) up to the highest concentration tested (19.25 μM), based on lactate dehydrogenase (LDH) release. Interestingly, the treatment of THP-1 macrophages with (lin-SB056-1)_2_-K at 19.25 μM in the absence of LPS resulted in a stronger cytotoxic effect (14% cell death) as compared to the cells co-cultured with both the peptide and LPS (5% cell death) (Figure 3b), suggesting a possible interplay between the two molecules with consequent sequestration of the peptide and reduced interaction with eukaryotic cells.

### 2.3. The Dimeric Derivative (Lin-SB056-1)_2_-K Exhibits Higher LPS-Binding Affinity than Its Monomeric Counterpart

**Figure 4:**
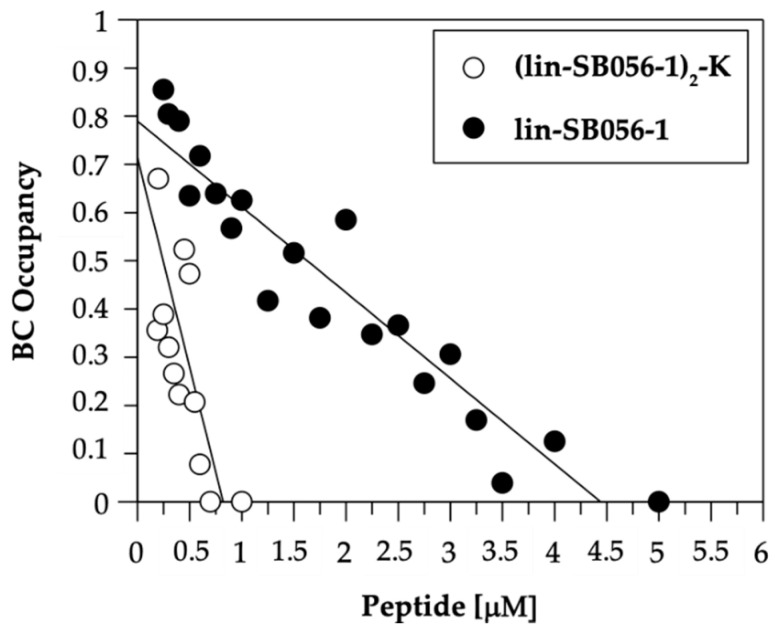
BC fluorescent displacement assay. Lin-SB056-1 and (lin-SB056-1)_2_-K bind to *P. aeruginosa* LPS causing the displacement of BC and the proportional dequenching of its fluorescence. [LPS]: 10 µg/mL; [BC]: 10 µM. Assay buffer: 50 mM Tris-HCl, pH 7.4. Data are reported as mean of three independent experiments, with standard deviation not exceeding 5%.

In order to get insights into the mechanisms underlying the anti-inflammatory effect of lin-SB056-1 and (lin-SB056-1)_2_-K, we initially evaluated their ability to interact with *P. aeruginosa* LPS. LPS-binding affinity of both lin-SB056-1 and its dendrimeric derivative (lin-SB056-1)_2_-K was monitored in a fluorometric assay that measures the competitive displacement of the BODIPY TR cadaverine (BC) fluorescent probe by the peptides [22]. As shown in Figure 4, both peptides bound to purified *P. aeruginosa* LPS and an incremental displacement of BC could be observed with increasing peptide concentrations in a micromolar range, thus confirming LPS interaction. The 50% LPS-binding affinity (Effective Displacement, ED_50_), defined as the peptide concentration needed to achieve displacement of half of the BC, corresponded to approximately 0.4 and 1.75 µM for the dendrimeric and the monomeric peptide, respectively. Hence, the dendrimeric form of the peptide displayed a significantly greater affinity for *P. aeruginosa* LPS compared to its monomeric form.

### 2.4. The Dimeric Derivative (Lin-SB056-1)_2_-K Exerts a Stronger LPS-Neutralizing Activity than Its Monomeric Counterpart

**Figure 5:**
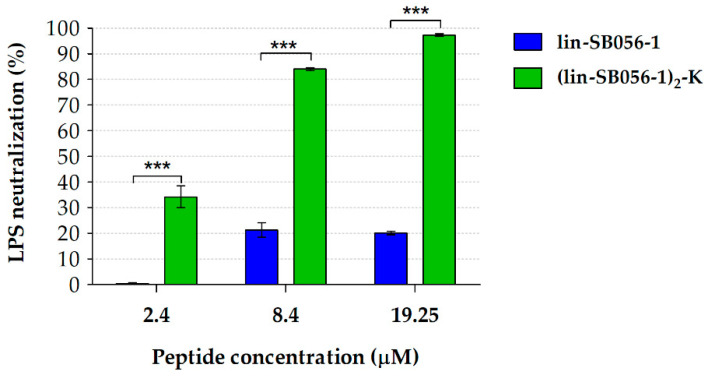
Neutralizing activity of lin-SB056-1 and (lin-SB056-1)_2_-K against *Escherichia coli* 011:B4 LPS (2 EU/mL) after 30 min of incubation (37 °C, shaking), assessed through a standard LAL test. LPS neutralization is expressed as percentage compared to the peptide-untreated control (i.e., free LPS). Data are reported as mean ± SEM of three independent experiments. *** *p* < 0.001 (Student’s *t*-test).

A standard *Limulus* Amebocyte Lysate (LAL) assay was employed to assess the ability of lin-SB056-1 and (lin-SB056-1)_2_-K to neutralize LPS activity. Due to its high sensitivity in detecting free LPS (i.e., pg/mL level) [23], the results of the test were used as an indirect indication of the amount of LPS sequestered and neutralized by the peptides. As shown in Figure 5, (lin-SB056-1)_2_-K demonstrated a higher LPS-binding ability than lin-SB056-1, resulting in the neutralization of almost 100% of the total LPS present in the assay at the concentration of 19.25 μM.

## 3. Discussion

The global issue of anti-microbial resistance coupled with the lack of new treatments for bacterial infections has stimulated interest in the use of AMPs for therapeutic purposes. Due to their unique mechanism(s) of action and wide spectrum of biological activities, AMPs are emerging as a promising alternative to conventional anti-microbial agents for the management of antibiotic-resistant and biofilm-associated infections [24,25]. Although mainly intended for topical use due to potential toxicity and reduced stability under physiological conditions, many different peptides with anti-microbial and/or immunomodulatory properties have already been tested for efficacy in clinical studies [26,27]. In this context, previous studies of our group focused on the evaluation of the anti-microbial properties of the semi-synthetic peptide lin-SB056-1, with particular attention to the development of strategies to improve its therapeutic potential against *P. aeruginosa* lung infections [17,18,19]. The peptide was found to exert promising anti-microbial activity, including against antibiotic-resistant strains and antibiotic-tolerant phenotypic variants (i.e., persister cells). Furthermore, it resulted to be effective against biofilms, showed low cytotoxicity towards mammalian cells and its activity was maintained under challenging host-mimicking conditions [17,18,28]. In particular, combination treatments of lin-SB056-1 with EDTA, and peptide dendrimerization emerged as valuable strategies to overcome the inhibitory effect of in vivo environmental factors (e.g., airway mucus and host cells) on peptide activity. When tested in in vivo-like models of lung infection, both the lin-SB056-1/EDTA combination and the dendrimeric derivative (lin-SB056-1)_2_-K were found to retain a remarkable anti-bacterial and anti-biofilm activity against *P. aeruginosa*, thereby representing promising candidates to be further developed [17,18].

In the present study, we evaluated whether lin-SB056-1 and (lin-SB056-1)_2_-K also showed anti-inflammatory properties, possibly resulting in a bifunctional mode of action and wider therapeutic possibilities. Inflammation represents a critical issue in many infectious diseases such as chronic lung infections caused by *P. aeruginosa* in CF patients. In such infections, the persistent inflammatory state associated with chronic immune stimulation by *P. aeruginosa* represents the major process leading to substantial deterioration of lung function and reduced life expectancy [29,30]. Therefore, the combined presence of anti-inflammatory and anti-microbial activities is highly desirable for effective therapies [7]. In order to assess the anti-inflammatory potential of lin-SB056-1 and (lin-SB056-1)_2_-K, we examined their ability to reduce the production of relevant inflammatory mediators (i.e., IL-1β, IL-6, IL-8 and TNF-α) that are massively secreted by lung epithelial cells and/or immune cells in response to *P. aeruginosa* infection and are commonly elevated in the airways of CF patients [31]. As major constituent of the outer membrane of Gram-negative bacteria and potent activator of the host immune response [32], LPS was selected as the main pro-inflammatory stimulus for the present study. Both purified (soluble) LPS and whole bacterial cells as source of membrane-associated LPS were employed for cell stimulation. Indeed, during the in vivo infection process, it is likely that LPS would be encountered by the host inflammatory cells in a bacterium-bound form as well as in various soluble forms (e.g., LPS released from bacteria following antibiotic treatment) [33,34], making it relevant the assessment of the anti-inflammatory potential of the peptides in both scenarios. Although comparable amounts of stimulus were used in the two experimental settings, differences in induction of cytokine production were observed between soluble LPS and whole *P. aeruginosa*. This discrepancy can be explained by taking into account the different ability of free and bacterium-bound LPS to interact with cellular effectors, and to the presence of additional pro-inflammatory components in intact bacterial cells [35,36]. Regardless of the tested pro-inflammatory agent and its stimulatory effect, the dendrimeric peptide (lin-SB056-1)_2_-K displayed a significant anti-inflammatory activity at relatively low, non-toxic concentrations. Analogously to the anti-microbial properties [18], (lin-SB056-1)_2_-K demonstrated a superior anti-inflammatory effect compared to its monomeric counterpart. At concentrations as low as 9.6 µM, it was able to reduce the levels of IL-1β, IL-6 and IL-8 in lung epithelial cells and/or macrophages stimulated with *P. aeruginosa* LPS. Notably, the same concentration of the peptide was also able to inhibit to some extent the pro-inflammatory response induced in epithelial cells by the whole bacterium. This finding suggests peptide’s ability to interact with components of the bacterial surface (e.g., LPS) and, in turn, affect the cell stimulatory capacity of the bacterium.

While the anti-microbial activities of AMPs are becoming increasingly understood, the mechanisms underlying their anti-inflammatory effect still need to be fully elucidated [37]. Nevertheless, the anti-inflammatory mode of action of many peptides has been attributed to their ability to efficiently bind LPS and/or block LPS recognition by effector cells [38,39,40]. For instance, the well-known human cathelicidin LL-37 has been shown to suppress LPS-induced pro-inflammatory responses in vitro and to protect from sepsis in animal models by sequestering soluble LPS [41]. Analogous LPS-neutralizing activities have been reported for other naturally-occurring AMPs (e.g., cecropins, lactoferrin and temporin) and AMP-based antibiotics (e.g., polymyxins) both in vitro and in vivo [42,43,44]. Considerable attention has also been devoted to the rational design of AMPs with enhanced LPS-binding affinity with the aim of boosting their anti-inflammatory properties [37,45,46]. Therefore, in order to elucidate whether lin-SB056-1 and (lin-SB056-1)_2_-K interact with LPS, we evaluated their capacity to bind *P. aeruginosa* LPS using a fluorescent displacement assay. In accordance with the peptide anti-inflammatory activity, the dendrimeric version of lin-SB056-1 displayed a stronger LPS-binding affinity in vitro than its monomeric counterpart. These observations correlated with the striking ability of (lin-SB056-1)_2_-K to neutralize the activity of LPS, as established in the LAL test. Indeed, differently from lin-SB056-1, the addition of the dendrimeric peptide to soluble LPS resulted in the reduction of LAL activity (i.e., inhibition of amebocyte lysate coagulation), most likely due to the formation of peptide/endotoxin complexes that prevented the binding of LPS to the *Limulus* anti-lipopolysaccharide factor. An indirect indication of the neutralizing interaction between the dendrimeric peptide and LPS was also provided by the cytotoxicity studies. In particular, the enhanced cytotoxicity of (lin-SB056-1)_2_-K towards THP-1 cells observed in the absence of LPS treatment suggested that the AMP/LPS interaction might have a role in the reduction of peptide binding to mammalian cells. Taken together, these findings pointed out the potential role of LPS sequestration by (lin-SB056-1)_2_-K in the suppression of LPS biological activity and contributed to explain the higher anti-inflammatory effect of the dendrimeric peptide compared to its monomeric counterpart.

It is generally thought that the dendrimeric architecture leads to improved biological activity due to a higher local concentration of the active sequence [47,48]. Possibly, thanks to its conformational plasticity, the dendrimeric organization might maximize target binding (e.g., LPS) through electrostatic interactions, leading to greater biological effects. Nevertheless, we cannot exclude a role of stability on the ability of dendrimeric peptides to establish solid interactions with LPS under specific experimental conditions. For instance, the stronger LPS-binding affinity of (lin-SB056-1)_2_-K compared to its monomeric counterpart could be explained by its lower susceptibility to the inhibitory effect of different experimental factors (e.g., salts, proteases, macromolecules and/or cellular components) on the establishment of electrostatic interactions [49,50]. Such peptide-LPS interactions could hamper LPS to assume an optimal folding for the binding to its receptors on effector cells with consequent reduction in the pro-inflammatory response [9,51]. Alternatively, as only monomeric forms of LPS are able to trigger cell activation, the dendrimeric peptide could act by inducing LPS aggregation. Although it has not been demonstrated for (lin-SB056-1)_2_-K, the inactivation of the pro-inflammatory activity of LPS through formation of supramolecular structures has been already documented for a number of AMPs [51,52,53,54]. Interestingly, there are not many examples of anti-microbial dendrimeric peptides able to bind LPS with high affinity. Gan and co-workers [55] have recently demonstrated the ability of the dendrimeric peptide G3KL [(KL)_8_(*K*KL)_4_(*K*KL)_2_*K*KL; *K* = branching lysine] to bind LPS and inhibit the LPS-induced release of TNF-α by macrophages. Conversely, the LPS-binding affinity of the SB041 dendrimer did not translate into LPS-neutralizing activity when the endotoxin was used to activate innate immune defense-like responses in RAW-Blue^TM^ macrophages [56].

## 4. Materials and Methods

### 4.1. Peptides

Lin-SB056-1 (KWKIRVRLSA-NH_2_) and (lin-SB056-1)_2_-K ([KWKIRVRLSA]_2_-K) were purchased from Peptide Synthetics (Fareham, England). Analysis of the synthetic peptides by high performance liquid chromatography and mass spectrometry revealed purity over 98%. Stock solutions were prepared by resuspending the lyophilized peptides in milli-Q water and stored at −80 °C.

### 4.2. Bacterial Strains and Culture Conditions

The reference strain *P. aeruginosa* PAO1 (ATCC 15692) was used in this study. For the preparation of stock cultures, bacteria were grown in Tryptone Soy Broth (TSB; Thermo Fisher Scientific, Waltham, MA, USA) until mid-exponential phase, then aliquoted and stored at −80 °C. For the preparation of the inoculum, a volume of 50 μL of the frozen culture was inoculated in 5 mL of TSB, and subsequently incubated for 18 h at 37 °C.

### 4.3. Cell Lines

Human non-small-cell lung adenocarcinoma A549 cells (ATCC CCL-185; LGC Standards, Milan, Italy) and human monocytic THP-1 cells (ATCC TIB-202; LGC Standards) were cultured in tissue culture flasks in Dulbecco’s modified essential medium (DMEM) (Euroclone, Milan, Italy) and RPMI 1640 medium (Euroclone), respectively. Both media were supplemented with 2 mM L-glutamine (Euroclone) and 10% heat-inactivated fetal bovine serum (FBS; Euroclone). Confluent monolayers were detached from the culture flask with 0.25% trypsin/1 mM EDTA solution (Euroclone), suspended in complete culture medium and seeded into flat-bottom 96-well plates at a final density of 1 × 10^4^ cells/well (A549) and 2 × 10^4^ cells/well (THP-1). A549 cells were cultured for 24 h at 37 °C, 5% CO_2_ in order to allow adhesion and formation of semi-confluent monolayers. THP-1 cells were cultured for 3 days (37 °C, 5% CO_2_) in the presence of 100 nM TPA (Sigma-Aldrich, St. Louis, MO, USA) to induce their differentiation into macrophage-like cells [57].

### 4.4. Cell Stimulation and Peptide Treatment

The anti-inflammatory potential of lin-SB056-1 and (lin-SB056-1)_2_-K was assessed by evaluating their ability to reduce LPS-induced production of pro-inflammatory cytokines by A549 cells and THP-1 macrophages. In order to ensure cytokine production, cell monolayers were stimulated with LPS from *P. aeruginosa* serotype 10 (purified by phenol extraction; Sigma-Aldrich, Milano, Italy) at a final concentration of 10 ng/mL (THP-1) and 800 ng/mL (A549). LPS concentrations used in the experiments were chosen based on preliminary assays aimed at identifying the minimum dose of the molecule able to stimulate cytokine production after 4 h of exposure. In order to assess the inhibitory activity of peptides on cytokine production, cell monolayers were incubated in complete culture medium in the presence of LPS and different concentrations of lin-SB056-1 and (lin-SB056-1)_2_-K (4.8 to 19.25 μM) for 4 h (37 °C, 5% CO_2_). Cells incubated in the presence of LPS alone were used as positive (cytokine production) control. Following incubation, cell culture supernatants were collected, filter sterilized (0.22 µm) and employed for the quantification of cytokines and the assessment of cell viability.

In some experiments, the ability of (lin-SB056-1)_2_-K to inhibit cytokine production by A549 cells stimulated with whole *P. aeruginosa* (PAO1 strain) was assessed. To this aim, *P. aeruginosa* PAO1 was grown in TSB until exponential phase, washed by centrifugation and resuspended in paraformaldehyde (4% solution in phosphate buffered saline, PBS) at a density of 1 × 10^8^ CFU/mL. Following fixation (30 min at 4 °C), bacteria were washed three times with complete DMEM and stored in aliquots at −20 °C until used. A549 cell monolayers were stimulated with fixed bacteria (5 × 10^6^ CFU/well/200 µL) in the presence and absence (cytokine production control) of different concentrations of (lin-SB056-1)_2_-K (9.6 and 19.25 μM) for 4 h (37 °C, 5% CO_2_). Following incubation, cell culture supernatants were collected as described above. Given that LPS represents approximately the 7.3% of the cell dry weight for *P. aeruginosa* PAO1 [58], the amount of membrane-bound LPS in the tested bacterial inoculum (approximately 500 ng/mL) is comparable to that of soluble LPS (i.e., 800 ng/mL) used to stimulate A549 cells.

### 4.5. Cytokine Assay

Different pro-inflammatory cytokines (i.e., IL-1β, IL-6, IL-8 and TNF-α) were quantified in cell culture supernatants through a bead-based multiplex immunoassay (LEGENDplex multi-analyte flow assay kit; BioLegend, San Diego, CA, USA) following manufacturer’s instructions. Briefly, 25 μL of cytokine standard or cell supernatant were incubated with 25 μL of anti-cytokine conjugated beads into V-bottom 96-well plates for 2 h. Following incubation, the plates were washed and subsequently incubated with the detection antibody (25 μL/well) for 1 h. A volume of 25 μL of streptavidin-phycoerythrin was added to each well, and the plates were incubated for another 30 min in the dark. Following consecutive washing steps, the beads were resuspended in the LEGENDplex wash buffer and the samples were analyzed by flow cytometry (BD Accuri C6 flow cytometer; BD Biosciences, Mountain View, CA, United States). Data were acquired with the BD Accuri C6 software (BD Biosciences) (300 events per analyte) and analyzed with the LEGENDplex software (BioLegend). Minimum detectable concentration (MDC+2STDEV) for the tested cytokines were as follows: IL-1β, 0.65 + 0.47 pg/mL; IL-6, 0.97 + 1.46 pg/mL; IL-8, 1.90 + 0.65 pg/mL; TNF-α, 0.88 + 0.27 pg/mL.

### 4.6. Cytotoxicity Assay

The cytotoxicity of lin-SB056-1 and (lin-SB056-1)_2_-K towards A549 and THP-1 cells was evaluated to exclude a possible correlation between reduction in cytokine levels and decrease in cell numbers. To this end, culture supernatants of cells stimulated with LPS in the presence of the peptides were employed to quantify the amount of cytoplasmic LDH released by dead cells. Cells incubated in culture medium alone or in the presence of LPS served as an indication of spontaneous cell death (cell viability controls), while cells treated with a commercial lysis buffer (Pierce LDH cytotoxicity assay kit, Thermo Fisher Scientific) were used as cell death control (100% cell lysis). The enzymatic activity of LDH in cell supernatants was measured using the Pierce LDH cytotoxicity assay kit (Thermo Fisher Scientific) following manufacturer’s instructions. The cytotoxic effect was determined as follows: cytotoxicity (%) = [(LDH activity of A/LDH activity of B) × 100], where A represents peptide-treated or untreated cells (both in the presence and absence of LPS), and B corresponds to the cell lysis control.

### 4.7. Fluorescent Displacement Assay

Measurement of the ability of lin-SB056-1 and its dendrimeric derivative to bind *P. aeruginosa* serotype 10 LPS (Sigma-Aldrich) in vitro was performed by a fluorescent displacement assay using the BODIPY TR cadaverine (BC) probe (5-(((4-(4,4-difluoro-5-(2-thienyl)-4-bora-3a,4a-diaza-s-indacene-3-yl)phenoxy)acetyl)amino)pentylamine, hydrochloride); Thermo Fisher Scientific), as described elsewhere [22]. BC binds to LPS by recognizing the lipid A portion and the probe fluorescence is quenched. When a peptide able to interact with LPS is added, BC is displaced from the complex and its fluorescence increases. LPS binding assays were carried out in 50 mM Tris-HCl buffer, pH 7.4. Aliquots of the peptides at a final concentration of 0.1 μM were successively added to a cuvette containing the LPS/BC complex (10 µg/mL *P. aeruginosa* LPS and 10 µM BC). Fluorescence measurements were performed with a Perkin-Elmer LS 55B spectrofluorometer (Perkin-Elmer, Waltham, MA, USA) using a thermostated (25 °C) cuvette apparatus (excitation: 580 nm; emission: 620 nm).

### 4.8. LAL Assay

The ability of lin-SB056-1 and (lin-SB056-1)_2_-K to neutralize the activity of LPS was analyzed using a standardized LAL assay (Pierce LAL chromogenic endotoxin quantitation kit; Thermo Fisher Scientific). Peptides at different concentrations (2.4 to 19.25 μM) were pre-incubated with *E. coli* 011:B4 LPS (2 Endotoxin Units (EU)/mL; EU = unit of measurement for endotoxin activity) for 30 min (37 °C, shaking) in order to promote the interaction between the two molecules. LPS alone was used as positive control. Following incubation, peptide-treated and untreated samples were mixed with the LAL reagent (1:1 ratio) and incubated for 10 min at 37 °C. Subsequently, 100 μL of the chromogenic substrate were added and incubated with the samples for 6 min. The reaction was terminated by adding 100 μL of acetic acid (25% v/v), and absorbance was measured at 405 nm (OD_450_) using a microplate reader (Multiscan FC, Life Technologies Italia, Monza, Italy). The amount of peptide-sequestered LPS was determined as follows: LPS neutralization (%) = [100 − (OD_peptide_/OD_positive control_ × 100)].

### 4.9. Statistical Analysis

All the experiments were performed at least three times, unless otherwise specified. Statistical analysis was carried out using SPSS statistics software, version 25 (SPSS, Chicago, IL, USA). Normal distribution of the data was verified using the Shapiro–Wilk test. Differences between mean values were evaluated with Student’s *t*-test for independent samples in case of two-sample comparison and one-way analysis of variance (ANOVA) followed by Tukey–Kramer post-hoc test in case of multiple comparison. A two-sample testing was carried out for cytotoxicity studies in order to analyze differences in the effect of the peptides (at the maximum concentration of 19.25 µM) used alone and in the presence of *P. aeruginosa* LPS. The same approach was exploited to compare the LPS-neutralizing activity of equimolar concentrations of lin-SB056-1 and (lin-SB056-1)_2_-K. Multiple comparisons were performed to highlight differences in cytokine production between cells treated with different concentrations of the peptides and the peptide-untreated control.

## 5. Conclusions

In conclusion, the dendrimeric derivative (lin-SB056-1)_2_-K emerged as attractive lead compound for the development of novel anti-microbials against *P. aeruginosa* chronic lung infections. Indeed, in addition to exerting a considerable anti-bacterial and anti-biofilm activity under challenging host-mimicking conditions, (lin-SB056-1)_2_-K was found to efficiently bind *P. aeruginosa* LPS and reduce LPS-stimulated pro-inflammatory cytokine release, thereby providing considerable potential for the control of pathological inflammatory responses associated with *P. aeruginosa* infections.
